# Involvement of N-6 Adenine-Specific DNA Methyltransferase 1 (*N6AMT1*) in Arsenic Biomethylation and Its Role in Arsenic-Induced Toxicity

**DOI:** 10.1289/ehp.1002733

**Published:** 2010-12-30

**Authors:** Xuefeng Ren, Maria Aleshin, William J. Jo, Russel Dills, David A. Kalman, Christopher D. Vulpe, Martyn T. Smith, Luoping Zhang

**Affiliations:** 1 Genes and Environment Laboratory, Division of Environmental Health Sciences, School of Public Health and; 2 Department of Nutritional Sciences and Toxicology, University of California–Berkeley, Berkeley, California, USA; 3 Department of Environmental and Occupational Health Sciences, School of Public Health and Community Medicine, University of Washington, Seattle, Washington, USA

**Keywords:** arsenic methylation, arsenic toxicity, arsenite, monomethylarsonous acid, N6AMT1

## Abstract

**Background:**

In humans, inorganic arsenic (iAs) is metabolized to methylated arsenical species in a multistep process mainly mediated by arsenic (+3 oxidation state) methyltransferase (AS3MT). Among these metabolites is monomethylarsonous acid (MMA^III^), the most toxic arsenic species. A recent study in *As3mt*-knockout mice suggests that unidentified methyltransferases could be involved in alternative iAs methylation pathways. We found that yeast deletion mutants lacking *MTQ2* were highly resistant to iAs exposure. The human ortholog of the yeast *MTQ2* is N-6 adenine-specific DNA methyltransferase 1 (*N6AMT1*), encoding a putative methyltransferase.

**Objective:**

We investigated the potential role of *N6AMT1* in arsenic-induced toxicity.

**Methods:**

We measured and compared the cytotoxicity induced by arsenicals and their metabolic profiles using inductively coupled plasma–mass spectrometry in UROtsa human urothelial cells with enhanced *N6AMT1* expression and UROtsa vector control cells treated with different concentrations of either iAs^III^ or MMA^III^.

**Results:**

*N6AMT1* was able to convert MMA^III^ to the less toxic dimethylarsonic acid (DMA) when overexpressed in UROtsa cells. The enhanced expression of *N6AMT1* in UROtsa cells decreased cytotoxicity of both iAs^III^ and MMA^III^. Moreover, *N6AMT1* is expressed in many human tissues at variable levels, although at levels lower than those of *AS3MT*, supporting a potential participation in arsenic metabolism *in vivo*.

**Conclusions:**

Considering that MMA^III^ is the most toxic arsenical, our data suggest that *N6AMT1* has a significant role in determining susceptibility to arsenic toxicity and carcinogenicity because of its specific activity in methylating MMA^III^ to DMA and other unknown mechanisms.

Inorganic arsenic (iAs) compounds are considered known human carcinogens that target multiple sites, including the lung, skin, and urinary bladder [International Agency for Research on Cancer (IARC) 2004; Pershagen 1981; Smith et al. 1992; Smith and Steinmaus 2009; Straif et al. 2009]. In addition, chronic exposure to high levels of iAs has been associated with the development of multiple diseases and deleterious health effects in humans (Abernathy et al. 1999; Kapaj et al. 2006).

In humans, as in many animal species, iAs is metabolized to monomethylarsonous acid (MMA) and dimethylarsonic acid (DMA). The most cited conceptual model of arsenic methylation involves the reduction of pentavalent iAs (iAs^V^) to trivalent iAs (iAs^III^), with subsequent methylation (Drobna et al. 2009). The general scheme is as follows:


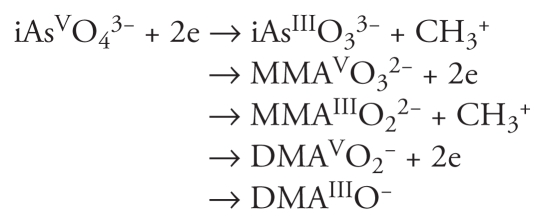


Among these metabolites, MMA^III^ is the most toxic arsenic species (Drobná et al. 2005; Ferrario et al. 2008; Kligerman et al. 2003; Petrick et al. 2001). It is generally accepted that arsenic (+3 oxidation state) methyltransferase (AS3MT) is responsible for catalyzing methyl group transfer from *S*-adenosyl methionine (SAM) to iAs (Thomas et al. 2007). However, a recent study by Drobna et al. (2009) showed that knockout of *As3mt* in the mouse does not completely abolish the methylation of iAs, suggesting that there are alternative pathways for arsenic methylation in these animals. Although Bentley and Chasteen (2002) and Hall et al. (1997) suggested that arsenic methylation could be due to gastrointestinal tract microbiota, they also speculated that unidentified methyltransferses may be responsible for the methylated arsenicals found in *As3mt*-knockout mice.

We conducted a genomewide, parallel phenotypic screen of yeast deletion mutants to identify the genes required for the growth of yeast in the presence of MMA^III^ and iAs^III^ (Jo et al. 2009). We found a yeast strain with deletion of *MTQ2*, which was highly resistant to iAs^III^. *MTQ2* encodes a SAM-dependent methyltransferase and has been shown to be involved in the methylation of release factor eRF1 in yeast (Polevoda et al. 2006). The human ortholog of the yeast *MTQ2* is N-6 adenine-specific DNA methyltransferase 1 (*N6AMT1*), a putative methyltransferase. Although the bacterial homologs of *N6AMT1* have been shown to methylate DNA N6-adenine (Stephens et al. 1996), the current data do not indicate its function in the methylation of adenine in the DNA of mammalian cells (Ratel et al. 2006).

Our goal in the present study was to explore the mechanism by which *N6AMT1* confers resistance to arsenic toxicity. We enhanced *N6AMT1* gene expression in UROtsa cells, given its relatively low expression in these cells. The UROtsa cell line, originally isolated from a primary culture of normal human uroepithelium, does not methylate arsenic because of the absence of *AS3MT* expression (Drobná et al. 2005; Styblo et al. 2000) and has been used as a model for bladder epithelium and arsenic-induced bladder cancer (Bredfeldt et al. 2006; Eblin et al. 2008; Sens et al. 2004). Here, we show that *N6AMT1* is a human methyltransferase specifically involved in the biomethylation of MMA^III^ to DMA. Given that MMA^III^ is the most toxic arsenical and its implication in arsenic toxicity and carcinogenicity, *N6AMT1* may have a significant role in modulating arsenic-induced toxicity and carcinogenicity.

## Materials and Methods

### Cultures of yeast strains and human UROtsa cells

The wild-type BY4743 yeast strain was purchased from Invitrogen (Carlsbad, CA), and the *MTQ2* deletion strain has the same background as the wild-type strain. Growth was conducted in rich media [yeast extract-peptone-dextrose (YPD)] at 30°C with shaking at 200 rpm. UROtsa cells (generously provided by P. Simeonova, National Institute for Occupational Safety and Health, Morgantown, WV) were cultured at a starting cell density of 4–5 × 10^4^ cells/mL in RPMI 1640 (Mediatech, Inc., Manassas, VA) with l-glutamine, 10% fetal bovine serum, 100 IU/mL penicillin, and 100 μg/mL streptomycin (Omega Scientific, San Diego, CA), under standard culturing conditions.

### Arsenical exposures

We purchased sodium arsenite [NaAsO_2_ (iAs^III^); purity > 99%] from Sigma-Aldrich (St. Louis, MO). Diiodomethylarsine [MMA^III^ iodide (MMA^III^)] was a generous gift from J. Gandolfi (University of Arizona, Tucson, AZ). iAs^III^ and MMA^III^ solutions were freshly prepared using sterile water (Milli-Q; Millipore, Billerica, MA) and protected from light before use. Yeast cells were treated with either iAs^III^ or MMA^III^ at concentrations ranging from 0 to 300 μM.Once UROtsa cells reached 70–80% confluence in culture, they were treated with iAs^III^ at concentrations from 0 to 100 μM or MMA^III^ at 0–5 μM.

### Yeast growth assay

Yeast strains were pregrown in YPD media to mid-log phase, diluted in fresh media to an optical density at 595 nm (OD_595_) of 0.0165, and inoculated into a 48-well microplate. Stock solutions of arsenicals were added to each culture with at least three replicate wells per dose.

Plates were incubated in a Tecan GENios spectrophotometer (Tecan Systems Inc., San Jose, CA) set to 30°C with intermittent shaking, and OD_595_ measurements were taken at 15-min intervals for 24 hr. Raw absorbance data were averaged for all replicates, corrected for background, and plotted as a function of time. The area under the curve (AUC) was calculated for the cultures in each well using Prism software (version 5.01; GraphPad Software, Inc., La Jolla, CA), and the treatments were averaged and expressed as a percentage of the control.

### Human tissue array and real-time quantitative polymerase chain reaction (PCR) assay

We used TaqMan-based real-time quantitative polymerase chain reaction (rt-qPCR) to quantify *N6AMT1* and *AS3MT* expression on a panel of 48 normal human tissues using the Human Rapid-Scan Plate (OriGene Technologies, Inc., Rockville, MD). The human tissues were selected from multiple individuals of different ethnicity and pooled together. We obtained the primers and probes used for amplification of *N6AMT1*, *AS3MT*, and *ACTB* (β-actin; control) from Applied Biosystems (Foster City, CA). Gene expression of *N6AMT1* and *AS3MT* was calculated relative to *ACTB* using the ΔΔ*C*_T_ method.

### *N6AMT1* gene expression vector constructs and stable cell lines

Human *N6AMT1* cDNA (GenBank accession no. NM_013240; National Center for Biotechnology Information 2011) was PCR amplified with primers 5′-AACGCAGCGAAGGACTAT-3′ and 5′-CAGTAGTTCTGGGCACAC-3′. The PCR product was gel purified (Qiagen, Valencia, CA) and cloned into pcDNA 2.1 vector (Invitrogen) according to the manufacturer’s instructions, and the sequence was confirmed. The pcDNA 2.1 vector containing the *N6AMT1* gene was excised using *Not*I/*Bam*HI restriction enzymes (New England Biolabs, Ipswich, MA) and subjected to gel purification. The nucleotides of the *N6AMT1* gene containing *Bam*HI and *Not*I overhangs were annealed and ligated to a linearized pRetro X-IRES-ZsGreen vector (Clonetech, Mountain View, CA) digested with *Bam*HI and *Not*I (New England Biolabs). The pRetro X-IRES-ZsGreen vector is a fluorescent retroviral expression vector that allows both a gene of interest and the ZsGreen gene to be expressed. The resultant constructs were amplified, purified, and sequenced. UROtsa cells were transfected with this constructed vector or a control vector using Lipofectamine 2000 reagent (Invitrogen) according to the manufacturer’s instructions. After incubation at 37°C for 8 hr, the supernatant fraction containing the retroviral vector was removed and replaced with normal growth medium. Cells grown for 48–72 hr were assessed by fluorescence microscopy. The ZsGreen fluorescent marker yields a bright green fluorescence, permitting direct monitoring of the delivery efficiency. Finally, the cell populations were sorted by the DAKO-Cytomation MoFlo High Speed Sorter (Dako North America, Carpinteria, CA), and the green fluorescent cells were purified and collected for continuing culture. The green fluorescent cells were used for additional experimentation.

### Semiquantitative reverse-transcription (RT)-PCR

UROtsa cells with either *N6AMT1* or plasmid vectors were collected, and total RNA was isolated from these cells using the Qiagen RNAEasy Mini kit. We performed a reverse transcription reaction using SuperScript II Reverse Transcriptase (Invitrogen) according to the manufacturer’s instructions. The PCR conditions for DNA amplification in the linear range were established on the GeneAmp PCR System 7600 (PerkinElmer, Inc., Wellesley, MA). The primers for DNA amplification were as follows: *N6AMT1*, 5′-AACGCAGCGAAGGACTAT-3′ and 5′-CAGTAGTTCTGGGCACAC-3′; *AS3MT*, 5′-GTGTCTGGGTGGTGCTTTATACTG-3′ and 5′-TGGAGGGCAGAACCCAATT-3′; and the housekeeping gene ACTB, 5-TCACCCACACTGTGCCCATCTACGA-3 and 5-CAGCGGAACCGCTCATTGCCAATGG-3. RT-PCR products were analyzed on 1% agarose gels.

### Cytotoxicity assay

We performed the 3-(4,5-dimethylthiazol-2-yl)-2,5-diphenyl-2H-tetrazolium bromide (MTT) assay to assess cell viability after arsenic treatment. Cells were cultured in 96-well plates in a volume of 100 μL medium/well at a density of 5 × 10^4^ cells/mL. Twenty-four hours after incubation with iAs^III^ or MMA^III^ (six replicates/arsenical concentration), 10 μL sterile MTT dye (Sigma-Aldrich; 5 mg/mL) was added to each well and plates were incubated at 37°C for 4 hr. The culture medium was then removed, and 200 μL dimethyl sulfoxide was added and thoroughly mixed for 10 min. Spectrophotometric absorbance at 570 nm was measured in a microplate reader.

### Arsenic species profile analysis by high-performance liquid chromatography/inductively coupled plasma–mass spectrometry (HPLC-ICP-MS) methods

UROtsa cells with *N6AMT1* and UROtsa cells with vector were grown in RPMI 1640 medium supplemented with 10% fetal bovine serum and antibiotics. Culture medium was collected after exposure to iAs^III^ or MMA^III^ for 24 hr or 3 days and stored at −80°C until analysis. Cells treated with iAs^III^ or MMA^III^ for 3 days were collected, lysed in RIPA buffer, and extracted in methanol by incubating overnight at 4°C in a rotational shaker. After centrifugation at maximum speed for 5 min at 4°C, supernatants were transferred to microcentrifuge tubes and stored at −80°C until analysis. Before analysis, samples were diluted 1:5 with water–methanol to bring the methanol concentration to 2.5%, incubated at 5°C to precipitate poorly soluble material, and filtered (0.45 μm). Analysis was performed by HPLC-ICP-MS (Agilent 1090 HPLC and Agilent 7500CE ICP-MS run in normal mode; both from Agilent Technologies, Santa Clara, CA) under conditions that resolved neutral, trivalent, and pentavalent iAs species. The ion-pairing method (Le et al. 2000) was used with major modifications to improve the resolution of the species. Briefly, calibrants were prepared from neat materials [As_2_O_3_ (Aldrich) and As_2_O_5_ (Acros), Sigma-Aldrich; DMA^V^ and MMA^V^ · 6H_2_O, Chem Service, West Chester, PA] in deionized water (≥ 18 MΩ). For speciation, we used a Phenomenex Gemini-NX column (3 μm, C18, 110Å, 150 × 4.6 mm; Torrance, CA) with a corresponding guard column at 40°C. Concentrations of arsenic species in stock solutions were standardized against NIST traceable commercial ICP-MS standards (VWR BDH Aristar Plus; Ultra Scientific, Kingstown, RI). Serial dilutions were made into deionized water. iAs^III^ and iAs^V^ species were quantified by separate calibrant series, and iAs^III^ concentration in the calibrants was corrected for any conversion to iAs^V^. The HPLC conditions were isocratic (5 mM tetrabutylammonium hydroxide, 10 mM ammonium carbonate, 2.5% methanol, pH 9.2, 1 mL/min) for 5 min; then a step gradient (5 mM tetrabutylammonium hydroxide, 30 mM ammonium carbonate, 2.5% methanol, pH 8.75, 1.2 mL/min) for 5 min to elute iAs^V^ was followed by step gradients (5 mM tetrabutylammonium hydroxide, 30 mM ammonium carbonate, 2.5% methanol, pH 9.2, 1.2 mL/min) for a 5-min equilibration to the initial pH and finally to the initial mobile phase for 5 min (1.2 mL/min). The data were analyzed by LC ChemStation A.09.03 and ICP-MS ChemStation B.03.03 software (Agilent Technologies).

### Data analysis

Statistical analyses were performed using one-way analysis of variance. Data represent mean ± SE of at least three independent experiments.

## Results

### Deletion of yeast MTQ2 leads to increased resistance to arsenic treatment

**Figure 1:**
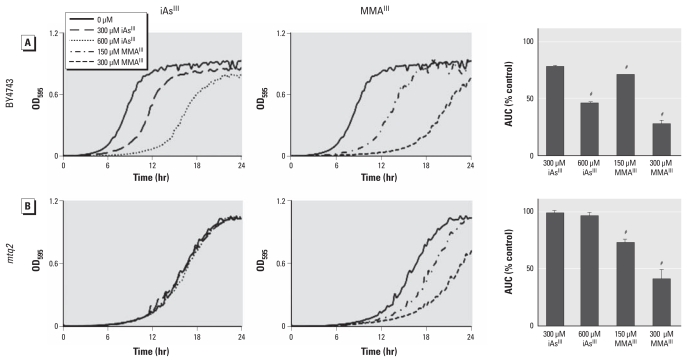
Deletion of the *MTQ2* gene in yeast results in increased resistance to arsenite (iAs^III^), shown as the growth phenotype of *MTQ2* mutant yeast cells (*B*) and the wild-type BY4743 cells (*A*) treated with 300 or 600 μM iAs^III^ or 150 or 300 μM MMA^III^. Growth curves show the OD_595_ for each treatment as a function of time for 24 hr. Bars represent the mean ± SE AUC for three technical replicates. At the doses tested, iAs^III^ treatment did not alter the growth pattern of *MTQ2* mutants but led to a dose-dependent reduction in growth of the wild-type strain; the growth patterns of both yeast strains were similar after MMA^III^ exposure. *^#^p*< 0.001, compared with control.

We evaluated the growth phenotype of the *MTQ2*-deletion mutants in the presence of either iAs^III^ or MMA^III^ (Figure 1). Deletion strains and their isogenic wild-type counterpart, BY4743, were treated with equioxic doses equivalent to the concentrations that resulted in 20% growth inhibition (IC_20_) and 2 × IC_20_, which were 300 and 600 μM for iAs^III^ and 150 and 300 μM for MMA^III^, respectively. iAs^III^ treatments had no effect on the growth of *MTQ2*-deletion mutants but significantly decreased growth of the wild-type strain. In comparison, the growth of both *MTQ2*-deletion mutants and wild-type yeast treated with MMA^III^ decreased to the same degree despite the higher toxicity of MMA^III^.

### Differential level of *N6AMT1* mRNA expression in human tissues

A direct search for sequence homology and conserved functional domains revealed that the human *N6AMT1* gene is orthologous to the yeast *MTQ2* gene. We used the web-based online tool Protein Function Prediction (PFP; Kihara Bioinformatics Laboratory 2010), to analyze and predict its potential functions (Hawkins et al. 2009). The suggested molecular functions of N6AMT1 include protein heterodimerization and methionine *S*-methyltransferase activity, with an almost certain (100%) predicted probability of being a methyltransferase. Given N6AMT1’s suggested function as a methyltransferase, we were interested in exploring its potential involvement in arsenic biomethylation. Considering that the primary methyltransferase responsible for arsenic metabolism in human cells is AS3MT, we did pairwise alignment analysis of N6AMT1 and AS3MT using EMBOSS Pairwise Alignment Algorithms, an online tool (European Bioinformatics Institute 2010). The two proteins shared about 25% similarity. Of the three sequence motifs found in most AS3MT homologs, only motif ILDLGSGSG is highly conserved in N6AMT1 [LEVGSGSG; see Supplemental Material, Figure 1(doi:10.1289/ehp.1002733)], whereas (D/N)PPY is present in N6AMT1 but not in AS3MT. These differences suggest that the mechanism by which N6AMT1 methylates arsenic may differ from that of AS3MT, if N6AMT1 is, in fact, involved in the methylation of arsenicals.

**Figure 2:**
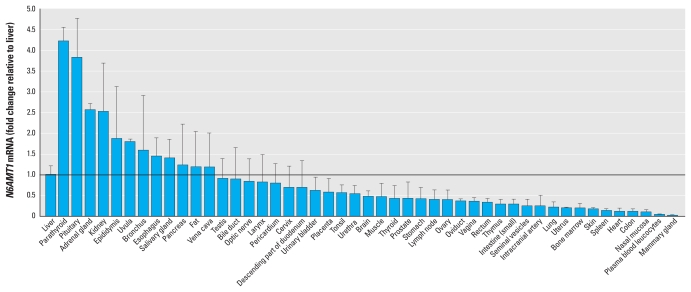
*N6AMT1* mRNA expression in human tissues, quantified by rt-qPCR analysis a panel of 48 normal human tissues; transcript levels of *N6AMT1* were normalized to *ACTB* expression and are shown as the fold change relative to liver.

A search of the Expressed Sequence Tags (EST) Database (National Center for Biotechnology Information 2010) revealed sequences matching the cDNA of *N6AMT1* in many human tissues with varied expression levels. To experimentally measure the mRNA expression of *N6AMT1* across tissues, we performed rt-qPCR analysis using cDNA from a panel of 48 human tissues contained in a tissue array (Figure 2). We confirmed the amplification products to be *N6AMT1* by DNA sequencing. Using the liver as a reference, the expression of *N6AMT1* was normalized to the expression level of *ACTB* [with cycle threshold (Ct) values ranging from 18 to 20] and found to be relatively highly expressed in tissues such as the parathyroid, pituitary, adrenal gland, and kidney, and weakly expressed in tissues such as the skin, lung, and mammary gland. We also measured *AS3MT* mRNA levels using the same tissue panel in order to compare *N6AMT1* expression in each tissue and *AS3MT* expression [see Supplemental Material, Figure 2 (doi:10.1289/ehp.1002733)]. The data showed that the level of *N6AMT1* mRNA was relatively low in most measured tissues compared with *AS3MT* mRNA. The presence of detectable levels of *N6AMT1* expression in all tissues analyzed suggests that this enzyme could be involved in methylating arsenicals. N6AMT1 may act in parallel to AS3MT and may be functional only under certain conditions or in certain tissues.

### Overexpression of *N6AMT1* in UROtsa cells increases resistance to arsenic treatment

**Figure 3:**
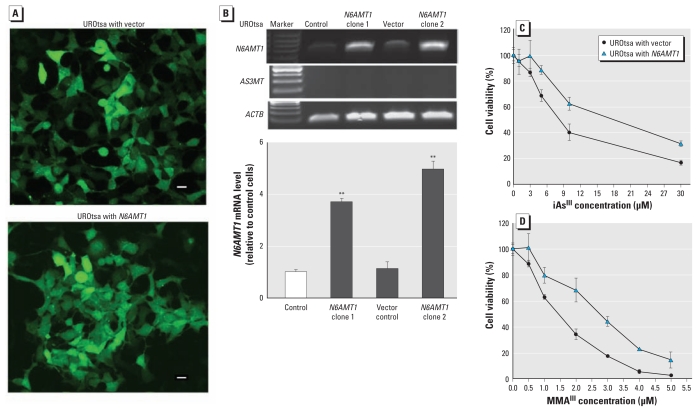
Enhancing expression of human *N6AMT1* in UROtsa cells increases resistance to both iAs^III^ and MMA^III^ treatment. (*A*) Representative images of UROtsa cells with either plasmid alone or plasmid with *N6AMT1;* bars = 10 μm. (*B*) Semiquantitative RT-PCR analysis shows dramatically increased *N6AMT1* expression in UROtsa cells with *N6AMT1* relative to control cells or cells with plasmid. *AS3MT* mRNA was not detected in either cell line; *ACTB* was used as the loading control. UROtsa cells with vector and with *N6AMT1* were treated with increasing concentrations of iAs^III^ (*C*) or MMA^III^ (*D*) for 24 hr, and cell viability was evaluated with MTT. Bars represent the mean ± SD of three independent experiments. Treatment with either iAs^III^ or MMA^III^ resulted in a dose-dependent decrease in viability. However, increased expression of *N6AMT1* in UROtsa cells led to resistance to both iAs^III^ (*C*) and MMA^III^ (*D*); this effect was more significant when cells were treated with MMA^III^ than with iAs^III^. ^**^p < 0.01, compared with control and vector control.

We also measured and compared the level of *N6AMT1* mRNA in several cell lines, including 293 (human embryonic kidney cells), HeLa, UROtsa, and HL60 (human promyelocytic leukemia cells). *N6AMT1* expression in UROtsa cells is relatively low, with a Ct value of about 33 (data not shown). This cell line also has almost no detectable level of *AS3MT*, making it an excellent model to study the role of *N6AMT1* in arsenic toxicity and metabolism in mammals. We enhanced *N6AMT1* expression in UROtsa cells using a retrovirus-based vector (Figure 3A) and found the level of *N6AMT1* mRNA in UROtsa cells to be significantly increased by approximately 5-fold in clone 2, as measured by semiquantitative RT-PCR (Figure 3B). We also measured *AS3MT* gene expression in these two cell lines and found no detectable mRNA level in either cell line (Figure 3B). We further confirmed these PCR results by real-time PCR analysis (data not shown). Unfortunately, we could not detect N6AMT1 protein levels in these cells using two commercially available antibodies. Transfected UROtsa cells did not have an altered doubling time or morphology in culture. The UROtsa cells with *N6AMT1* (*N6AMT1*-enhanced cells) and the UROtsa cells with vector cells (vector control) were treated with either iAs^III^ or MMA^III^ at a series of concentrations for 24 hr. Arsenical treatments induced a dose-dependent decrease in viability of both cell lines. However, increased expression of *N6AMT1* in UROtsa cells resulted in higher viability after iAs^III^ and MMA^III^ treatment at almost all concentrations tested, compared with the UROtsa vector control cells (Figure 3C,D). This increased arsenic resistance was more apparent in cultures treated with MMA^III^ than with iAs^III^, approximately 2- and 1.3-fold, respectively.

### Enhanced N6AMT1 in UROtsa cells methylates MMA^III^ to DMA

We collected medium and cell extracts from cultures of UROtsa cells with *N6AMT1* and UROtsa cells with vector treated with different concentrations of either iAs^III^ or MMA^III^ for up to 3 days and then measured and analyzed arsenic metabolic profiles using ICP-MS [for representative chromatograms, see Supplemental Material, Figure 3 (doi:10.1289/ehp.1002733)]. Methylated metabolites were undetectable either in the media (Table 1) or in the cell extract (Table 2) from cultures of UROtsa cells with *N6AMT1* or of UROtsa cells with vector, after treatment of with iAs^III^, suggesting that N6AMT1 does not methylate iAs^III^. When UROtsa cells with vector control were treated with MMA^III^, levels of MMA^V^ but not dimethylarsinic acid (DMA^V^) increased in the media (Table 3) and cells (Table 2). Treatment with MMA^III^ of UROtsa cells with *N6AMT1* resulted in similarly increased levels of MMA^V^ andled to increased levels of DMA^V^ in the media after 24 and 72 hr (Table 3) and, to a lesser degree, in the cell extracts after 72 hr (Table 2). At 24 hr, the level of DMA^V^ was similar at each dose level, but after 3 days treatment levels in both media and cell extract increased in relation to the initial concentration of MMA^III^, in a dose-dependent manner. Moreover, the amount of DMA^V^ in the culture medium increased 5-fold after 3 days of treatment (1 μM MMA^III^) compared with 1 day of treatment.

## Discussion

### MMA^III^ is the most toxic arsenic metabolite *in vivo* and *in vitro*

In humans, iAs is metabolized to methylated arsenical species in a multistep process. Methylated arsenicals, especially MMA^III^, may be more toxic than iAs both *in vivo* and *in vitro* (Drobná et al. 2005; Ferrario et al. 2008; Kligerman et al. 2003; Petrick et al. 2001). In cultured human cells, MMA^III^ is the most toxic arsenical (Drobná et al. 2005; Ferrario et al. 2008; Petrick et al. 2001) and inhibits several key cellular proteins, such as glutathione reductase (Styblo et al. 1997) and thioredoxin reductase (Lin et al. 1999, 2001). Several studies have shown that MMA^III^ is capable of inducing genetic damage and changes in signal transduction by either direct or indirect mechanisms (Ahmad et al. 2002; Kligerman et al. 2003; Nesnow et al. 2002). In addition, exposure to MMA^III^ for 52 weeks induced malignant transformation of UROtsa cells (Bredfeldt et al. 2006). Epidemiological studies have suggested that individuals who excrete a higher proportion of ingested arsenic as MMA^III^ are more susceptible to arsenic-related cancer (Steinmaus et al. 2006). MMA^III^ has been proposed as the ultimate genotoxic form of arsenic (Kligerman et al. 2003), and the existing evidence indicates that biomethylation of iAs to MMA^III^ is likely to alter the adverse effects of environmental arsenic exposure on human health.

### AS3MT is primarily responsible for methylating iAs to MMA^III^ and DMA in humans

The arsenic methyltransferase AS3MT is recognized as the primary enzyme responsible for conversion of iAs to its methylated metabolites MMA^III^ and DMA (Lin et al. 2002; Wood et al. 2006). Studies have shown that single-nucleotide polymorphisms in *AS3MT* lead to different urinary arsenical profiles (Agusa et al. 2009; Schläwicke Engström et al. 2009; Wood et al. 2006), some of which are associated with increased risk of premalignant skin lesions (Valenzuela et al. 2009). Although these data suggest that AS3MT plays a critical role in arsenic methylation and toxicity, a recent study showed that *As3mt*-knockout mice retain some ability to methylate arsenicals, suggesting the existence of other methyltransferases that could be involved in alternative arsenic metabolism pathways (Drobna et al. 2009).

### N6AMT1 is capable of methylating MMA^III^ to DMA

In this study, we found that N6AMT1 has the capacity to methylate MMA^III^ to DMA. The expression of *N6AMT1* is generally low compared with the expression level of *AS3MT* in most human tissues. In addition, the low sequence homology shared between these two proteins, about 25%, supports differences in substrate specificity and, possibly, in mechanisms of arsenic methylation. In contrast to AS3MT, which methylates iAs to the more toxic MMA^III^, N6AMT1 methylates MMA^III^ to the less toxic DMA. This is consistent with the increased resistance to MMA^III^ of UROtsa cells overexpressing *N6AMT1* compared with vector control cells, but other mechanisms likely also contribute to this increased resistance. Thus, our results suggest that *N6AMT1* may play a role in modulating arsenical-induced toxicity and that decreased *N6AMT1* expression or activity could have a significant impact on arsenic-induced toxicity and perhaps carcinogenicity under certain conditions or in certain tissues.

### N6AMT1 in human cells responded to arsenicals differently from MTQ2 in yeast

We noted differences in response to arsenical treatments between yeast and human cells. Specifically, the *MTQ2*-deletion yeast strain is resistant only to iAs^III^. We did not find evidence of iAs^III^ methylation in yeast wild-type; that is, DMA and MMA levels in cells and culture media were below the limit of quantitation (data not shown). Therefore, the function of *MTQ2* in arsenic toxicity may not be related to iAs^III^ methylation. In contrast, overexpression of *N6AMT1* in UROtsa cells leads to resistance to both iAs^III^ and MMA^III^, which provides evidence of its involvement in protection from these arsenicals. These results indicate that the orthologous genes—*MTQ2* in yeast and *N6AMT1* in humans—have different roles in the cellular response to arsenic toxicity.

### Conversion of MMA^III^ to DMA by N6AMT1 needs to be further confirmed biochemically

Our analyses showed that enhanced expression of *N6AMT1* in UROtsa cells converts MMA to DMA. However, purified recombinant N6AMT1, in the presence of SAM and other cofactors, was unable to methylate iAs^III^ or MMA^III^ (data not shown). N6AMT1 dimerizes with tRNA methyltransferase 11-2 homolog (TRMT112), which appears to be necessary for proper N6AMT1 function (Figaro et al. 2008) and is consistent with heterodimerization activity as predicted with PFP. Thus, the lack of N6AMT1-dependent MMA^III^ methylation in this test tube experiment may be due to the absence of TRMT112 or another unknown protein. In addition, *N6AMT1* overexpression increased resistance to iAs^III^, although UROtsa cells were not able to methylate iAs^III^ to MMA^III^ or to other methylated species, suggesting that mechanisms other than participation in arsenic methylation might be involved. It is not clear whether interaction between N6AMT1 and TRMT112 has a role in arsenic toxicity, but it is certainly worthy of further investigation.

## Conclusions

Our data suggest an important potential role of *N6AMT1* in modulating arsenical-induced toxicity by methylating MMA^III^ to the less toxic DMA. However, further investigation is warranted to determine whether N6AMT1 can methylate MMA^III^ *in vivo* and also to identify the genetic and environmental factors that can alter *N6AMT1* expression and/or activity. Our ongoing experiments are focused on the biochemical characterization of N6AMT1, specifically its capacity to methylate MMA^III^, as well as its ability to modulate arsenic toxicity and carcinogenicity.

## Figures and Tables

**Table 1:** Arsenic metabolic profile in cell culture medium after iAs^III^ treatment of UROtsa cells with vector and UROtsa cells with *N6AMT1* (ng/mL; mean ± SE).

	UROtsa cells with vector				UROtsa cells with *N6AMT1*			
iAs^III^ (μM)	iAs^(III +V)^	MMA^III^	MMA^V^	DMA^V^	iAs^(III +V)^	MMA^III^	MMA^V^	DMA^V^
1 day								
0	BLOQ	BLOQ	BLOQ	BLOQ	BLOQ	BLOQ	BLOQ	BLOQ
1	261.50 ± 126.22	BLOQ	BLOQ	BLOQ	417.00 ± 1.41	BLOQ	BLOQ	BLOQ
3	1109.00 ± 11.31	BLOQ	BLOQ	BLOQ	1159.00 ± 12.02	BLOQ	BLOQ	BLOQ
10	3695.50 ± 10.96	BLOQ	BLOQ	BLOQ	3751.00 ± 72.12	BLOQ	BLOQ	BLOQ

3 days								
0	BLOQ	BLOQ	BLOQ	BLOQ	BLOQ	BLOQ	BLOQ	BLOQ
1	425.50 ± 8.13	BLOQ	BLOQ	BLOQ	414.50 ± 5.30	BLOQ	BLOQ	BLOQ
3	1034.00 ± 2.12	BLOQ	BLOQ	BLOQ	1017.00 ± 7.07	BLOQ	BLOQ	BLOQ

BLOQ, below the limit of quantitation (the *m*/*z* 75 signal at the retention time of the species is not distinguishable from the baseline signal).

**Table 2:** Arsenic metabolic profile in cell extract after 3-day iAs^III^ and MMA^III^ treatment of UROtsa cells with vector and UROtsa cells with N6AMT1 (ng/mL; mean ± SE).

	UROtsa cells with vector				UROtsa cells with N6AMT1			
Treatment (μM)	iAs^(III+V)^	MMA^III^	MMA^V^	DMA^V^	iAs^(III+V)^	MMA^III^	MMA^V^	DMA^V^
iAs^III^								
0	BLOQ	BLOQ	BLOQ	BLOQ	BLOQ	BLOQ	BLOQ	BLOQ
1	2.7 ± 0.3	BLOQ	BLOQ	BLOQ	5.7 ± 0.6	BLOQ	BLOQ	BLOQ
3	12.0 ± 2.0	BLOQ	BLOQ	BLOQ	14.5 ± 0.5	BLOQ	BLOQ	BLOQ

MMA^III^								
0	BLOQ	BLOQ	BLOQ	BLOQ	BLOQ	BLOQ	BLOQ	BLOQ
0.5	BLOQ	BLOQ	3.5 ± 0.7	BLOQ	BLOQ	BLOQ	3.0 ± 0.1	2.8 ± 0.2
1	BLOQ	BLOQ	2.9 ± 0.8	BLOQ	BLOQ	BLOQ	4.7 ± 0.0	4.8 ± 0.6

BLOQ, below the limit of quantitation (the *m*/*z* 75 signal at the retention time of the species is not distinguishable from the baseline signal).

**Table 3:** Arsenic metabolic profile in cell culture medium after MMA^III^ treatment of UROtsa cells with vector and UROtsa cells with N6AMT1 (ng/mL; mean ± SE).

	UROtsa cells with vector				UROtsa cells with *N6AMT1*			
MMA^III^ (μM)	iAs^(III+V)^	MMA^III^	MMA^V^	DMA^V^	iAs^(III+V)^	MMA^III^	MMA^V^	DMA^V^
Treated 1 day								
0	BLOQ	BLOQ	BLOQ	BLOQ	BLOQ	BLOQ	BLOQ	BLOQ
0.5	BLOQ	Present	112.00 ± 0.71	BLOQ	BLOQ	Present	104.00 ± 0.71	21.50 ± 0.35
1	BLOQ	Present	236.50 ± 15.20	BLOQ	BLOQ	Present	210.00 ± 2.12	22.50 ± 1.06
2	BLOQ	Present	468.00 ± 17.68	BLOQ	BLOQ	Present	424.50 ± 6.72	20.00 ± 0.71

Treated 3 days								
0	BLOQ	BLOQ	BLOQ	BLOQ	BLOQ	BLOQ	BLOQ	BLOQ
0.5	BLOQ	Present	115.00 ± 2.12	BLOQ	BLOQ	Present	106.00 ± 2.83	72.00 ± 4.24
1	BLOQ	Present	277.00 ± 5.66	BLOQ	BLOQ	Present	236.50 ± 9.55	105.50 ± 4.60

BLOQ, below the limit of quantitation (the *m*/*z* 75 signal at the retention time of the species is not distinguishable from the baseline signal). “Present” indicates that the peak was seen at MMA^III^ retention time; no concentration is given because the samples were not analyzed by the MMA^III^ assay.
